# When to stop: Transfusions, difficult conversations and creativity

**DOI:** 10.1002/hem3.79

**Published:** 2024-05-28

**Authors:** Sophie Evans, Stephen P. Hibbs

**Affiliations:** ^1^ North Bristol NHS Trust Bristol UK; ^2^ Wolfson Institute of Population Health Queen Mary University of London London UK

*The prevailing story of haematology is of progress and advancement. If one treatment doesn't work, we have others up our sleeve – or perhaps just a year or two away from another breakthrough drug. But this dominant optimism does not reflect the options available to every patient entering our clinics. Frail elders with myelodysplastic syndrome are offered “supportive care”, primarily through regular blood transfusions. Clinicians often recognize that a time comes when the burdens of transfusions seem to outweigh their benefits, in our understanding of an individual's situation. But these burdens and benefits are deeply personal to an individual patient. Weighing them up is freighted with the fear of dying and potential accusations of “giving up”, and clinicians may choose to avoid discussing the question of whether to continue*.

*Here, Dr. Sophie Evans unpacks two creative approaches designed to open up understanding in this difficult scenario. Sophie explores her own observations and questions from clinical encounters by developing these into short visual stories. Then, by inviting patients to undertake a creative writing exercise, she gives them permission to express thoughts that may be difficult to say explicitly to their clinicians – or even to themselves. Her creative approaches highlight the value of bringing our own humanity and creativity to the conversations we find hardest in haematology practice – like when to stop*.

— Stephen Hibbs, Scientific Editor

When working within a haematology day unit I met patients receiving regular blood products as part of a ‘best supportive care’ approach. For some, this was because their disease had not responded to rounds of treatment; others had comorbidities or frailty that made chemotherapy inappropriate. I was often presented with a stack of blood product prescription charts to fill in for the following day. Often I had never met the patients in question, and sometimes there was no documentation about their transfusion regime, or most importantly about the aims of transfusion. For some patients having best supportive care for conditions like myelodysplastic syndrome, myelofibrosis, or aplastic anaemia, there are no prescribing guidelines, and the goal of treatment may never have been discussed. The easiest thing, especially when I was multitasking and under pressure to make quick decisions, was to repeat the previous prescription.

But as I got to know particular individuals, questions began to arise in my mind. An elderly patient called Bill* had been attending the day unit every 2 weeks for blood transfusions to relieve symptoms of anaemia, caused by myelodysplastic syndrome. Bill had tried oral chemotherapy but had suffered severe infections requiring hospitalisation as a result, and so his treatment had been stopped. Initially, his fatigue and breathlessness were managed well with transfusions every month, but within 6 months, he found the symptom relief would wane after just a week, leaving him lethargic. Accumulation of excess iron from blood transfusions added to his symptom burden. He went from walking into the day unit looking upbeat to being wheeled in looking withdrawn. I started to wonder whether his treatment made him feel better. How could we measure this? Was it right to keep giving him blood transfusions if they didn't help him feel better?

Since medical school, I have used art and comics to reflect. I have been drawn to patient stories in the realm of graphic medicine, using art and cartoons to explore illness and health narratives. These visual representations are unique reflections in that they allow me to view the artist's perspective in a way that prose does not. Creating something like a comic also allows me time to order my thoughts, to orient and contextualise experiences, and to assign meaning to them. Below is a depiction of an interaction I had with Bill**:


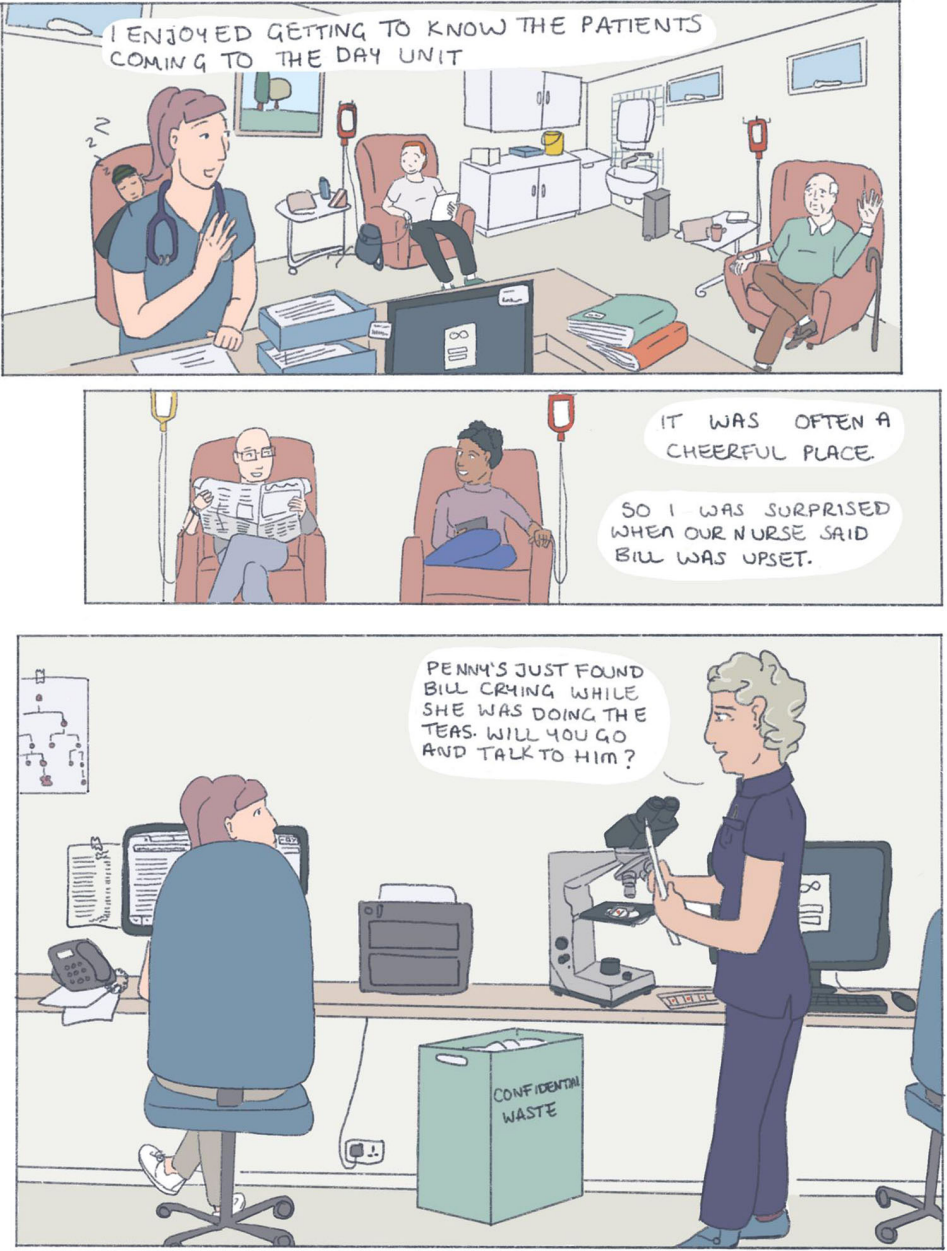



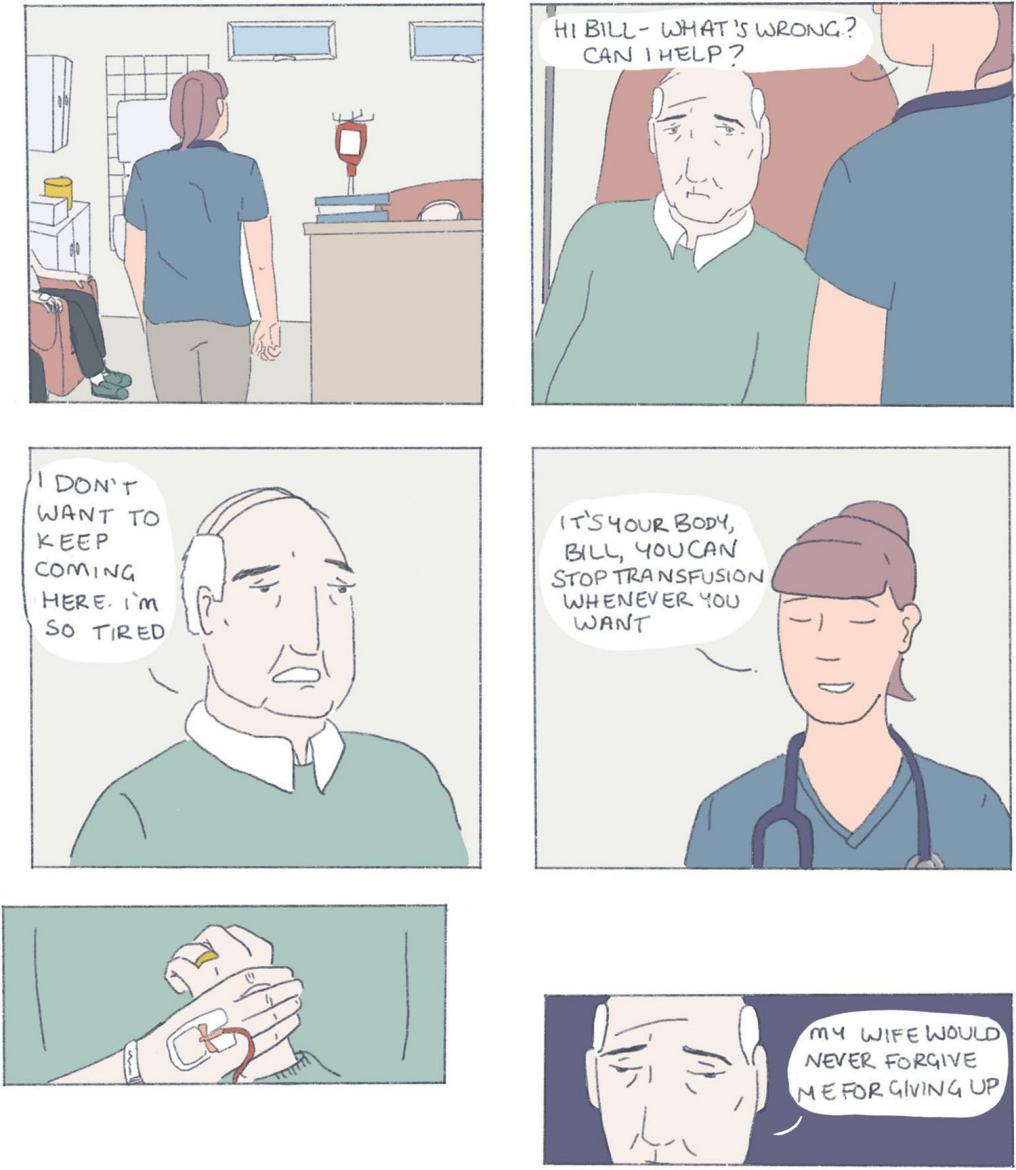



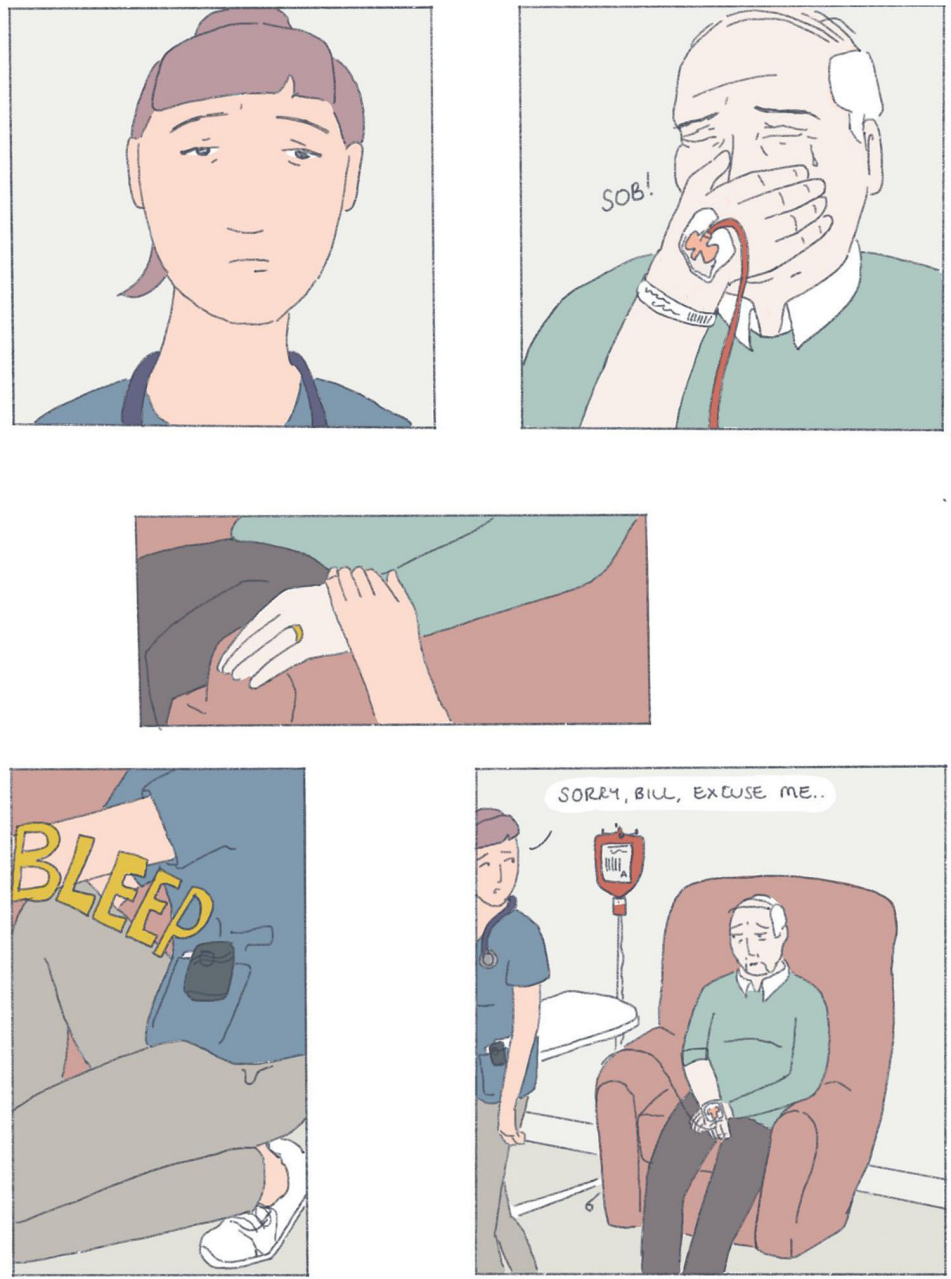


When I revisit and reflect on this comic, I am clear that Bill needed the time and space to explore his feelings and motivations for treatment. My suggestion of a simple binary choice—to stop or to continue transfusions—looked immature and ill‐considered when Bill opened up to me. Without knowing ‘the right thing' to say I chose to sit with him until my pager inevitably went off. I felt that I let him down by not giving him an answer or a plan. Bill had clearly expressed his misery in the hours spent at the day unit, but did I have the right to suggest that he stop attending?

For many of the other patients on the day unit, the horizon they look to is either cure or death—and this uncertainty brings its own challenges. But for patients like Bill, their outlook is between death or ‘artificially sustained life’. In a group of dialysis‐dependent patients interviewed about their quality of life, themes of reflection included the loss of meaningful time to the burden of dialysis treatment, the inability to process the end of life while still receiving treatment, and, surprisingly, a feeling that they had not chosen to start dialysis even though they may have attended preparatory consultations about it.^1^ On this last point, the patients felt they were choosing life as opposed to choosing dialysis. As a doctor, I must be aware of the impact that these intertwined decisions have on a patient giving informed consent. Over time, patients' direct experience allows them to understand more about the treatment decisions they made prior to commencing. Revisiting what treatment means for them provides a space to decide if they wish to continue.

Discussing treatments and medical conditions can feel abstract. I invited patients to the day unit to try a short creative writing exercise. I gave out a writing prompt of ‘What have you learned from living with your condition?’. Several anonymous responses were returned to the adapted shoebox I left on the reception desk. I was touched to hear from the nurses that some patients had kept their writing to themselves.

A range of accounts were included in the responses, from a young adult who felt restricted by their condition and physical ability compared to their peers, to an older patient who had received several unusual diagnoses since childhood and had assimilated hospital care into their routine. An elderly patient claimed their condition ‘was not the first thing [I] think about on waking up in the morning’. It struck me that there seemed to be an adjustment in patient narrative over time living with their conditions. This adjustment was not a passive process. The act of reflection and of finding meaning in change can help individuals to shape their stories. Clinicians can create space for patients through direct discussion as well as creative means such as suggesting keeping a diary, writing poetry, or drawing.

The decision of when to stop is not mine to make. Further clinical evidence might give patients more information about their options, but will not give an ‘answer’ either. By giving permission to patients like Bill to discuss these decisions with trusted clinicians, we challenge the implicit message that treatment must continue and that they must be booked for their next transfusion at the end of each appointment. I wonder if opportunities to let patients explore and shape their illness stories might help them find a way through when treatments are limited and evidence is lacking.

## AUTHOR CONTRIBUTIONS

Both authors conceptualised the article. Sophie Evans wrote the initial draft and authored the figures. Stephen P. Hibbs critically reviewed the article and revised it. All authors agreed to the final version.

## CONFLICT OF INTEREST STATEMENT

The authors declare no conflict of interest.

## FUNDING

SPH is supported by a HARP doctoral research fellowship, funded by the Wellcome Trust (Grant number 223500/Z/21/Z). No funding was received for this publication.

## Data Availability

Data sharing is not applicable to this article as no datasets were generated or analyzed during the current study.

## FOOTNOTE

* Names and details about the cases discussed have been changed to prevent identification. Any resemblance to living people in the illustrations of patients is coincidental.

** Comic illustration and messaging created and authored by Sophie Evans, the manuscript's first author.
